# Vector and Host C-Type Lectin Receptor (CLR)–Fc Fusion Proteins as a Cross-Species Comparative Approach to Screen for CLR–Rift Valley Fever Virus Interactions

**DOI:** 10.3390/ijms23063243

**Published:** 2022-03-17

**Authors:** Kathleen Schön, Dimitri L. Lindenwald, João T. Monteiro, Julien Glanz, Klaus Jung, Stefanie C. Becker, Bernd Lepenies

**Affiliations:** 1 Institute for Parasitology & Research Center for Emerging Infections and Zoonoses, University of Veterinary Medicine Hannover, 30559 Hanover, Germany; kathleen.schoen@tiho-hannover.de; 2 Institute for Immunology & Research Center for Emerging Infections and Zoonoses, University of Veterinary Medicine Hannover, 30559 Hanover, Germany; dimitril@gmx.net (D.L.L.); terenomonteiro.joao@mh-hannover.de (J.T.M.); 3 Institute for Animal Breeding and Genetics, University of Veterinary Medicine Hannover, 30559 Hanover, Germany; julien.glanz@lazbw.bwl.de (J.G.); klaus.jung@tiho-hannover.de (K.J.)

**Keywords:** Rift Valley fever virus, C-type lectin receptors, *Aedes aegypti*, C-type lectin domain-containing proteins, fusion proteins

## Abstract

Rift Valley fever virus (RVFV) is a mosquito-borne bunyavirus endemic to Africa and the Arabian Peninsula, which causes diseases in humans and livestock. C-type lectin receptors (CLRs) represent a superfamily of pattern recognition receptors that were reported to interact with diverse viruses and contribute to antiviral immune responses but may also act as attachment factors or entry receptors in diverse species. Human DC-SIGN and L-SIGN are known to interact with RVFV and to facilitate viral host cell entry, but the roles of further host and vector CLRs are still unknown. In this study, we present a CLR–Fc fusion protein library to screen RVFV–CLR interaction in a cross-species approach and identified novel murine, ovine, and *Aedes aegypti* RVFV candidate receptors. Furthermore, cross-species CLR binding studies enabled observations of the differences and similarities in binding preferences of RVFV between mammalian CLR homologues, as well as more distant vector/host CLRs.

## 1. Introduction

Hundreds of arthropod-borne viruses (acronym: arboviruses) such as Zika virus, Dengue virus, or Rift Valley fever virus (RVFV) are transmitted to humans via the bite of infected mosquitos. They cause severe diseases or even death in endemic areas [1,2]. RVFV (Order *Bunyavirales*, Family *Phenuiviridae* [3]), as one of these arboviruses, is endemic to Africa and the Arabian Peninsula [4,5]. Besides public health issues in humans, Rift Valley fever poses a major threat to livestock and agricultural productivity. Sheep and goats are among the most susceptible farm animals [6]. After experimental infection, the mortality rate of newborn lambs and the abortion rate of pregnant ewes reached nearly 100% [7,8]. Consequently, periodically occurrent Rift Valley fever outbreaks result in high economic losses, in addition to illnesses and deaths.

Similar to other bunyaviruses, RVFV is enveloped with a tri-segmented single-stranded RNA genome, which replicates in the host cell cytosol [9]. The smallest RNA segment (S) encodes for a nucleoprotein and a nonstructural protein (NSs), while the largest (L) encodes for the RNA-dependent RNA polymerase [9,10]. The medium RNA segment (M) encodes a 78 kDa protein, a second nonstructural protein (NSm), and two envelope glycoproteins, Gn and Gc [11,12]. During the initial step of virus entry, the surface glycoproteins are primary targets for pattern recognition receptors (PRRs) [8,13]. PRRs are transmembrane or soluble receptors that recognise evolutionarily conserved ligands at the pathogens’ surface, the so-called pathogen-associated molecular patterns [14]. Besides Toll-like receptors, RIG-I-like receptors, or NOD-like receptors, C-type lectin receptors (CLRs) are a major class of PRRs [14]. CLRs bind their ligands, mainly carbohydrates, with a specific carbohydrate recognition domain (CRD) [15]. As complex as the morphology of CLRs in mammalians can be, their functionality is as diverse as they can be. CLRs expressed by myeloid cells play important roles in innate immunity [16,17]. These myeloid CLRs recognise pathogen-associated glycoconjugates to elicit immune responses by stimulating the production of cytokines or chemokines or by inducing phagocytosis [16,17]. Myeloid DAP-12-associating lectin (MDL-1, also known as CLEC5A), as one example, is present on monocytes, macrophages, and neutrophils [18] and activates the release of pro-inflammatory cytokines, which induce the severe inflammatory reaction in Dengue virus patients [19,20]. While some CLRs such as MDL-1 induce antiviral responses, others can facilitate viral entry. Human DC-SIGN, a type II transmembrane protein mainly expressed by immature and mature dendritic cells [21,22], is exploited as an endocytic receptor by influenza A virus and phleboviruses, such as Uukuniemi virus and RVFV [23,24]. More recently, DC-SIGN was reported as attachment receptor for SARS-CoV-2, which enhances the ACE2 receptor-dependent infection of host cells [25,26].

Since the first decoding of the RVFV vector *Aedes aegypti* genome in 2007, 52 C-type lectin domain-containing proteins (CTLDcps) have been found [27,28]. Some of them were already reported to be involved in viral transmission [29,30,31]. Furthermore, some CTLDcps genes are up- or downregulated during viral infection and may therefore be involved in early or late viral vector response [29,32]. However, there is a knowledge gap regarding the role of mosquito CTLDcps in RVFV infection.

To identify potential CLR–RVFV interactions in a One Health approach, we employed murine and ovine CLR–human Fc (hFc) fusion protein libraries [33,34,35] and extended them with CLR–hFc fusion proteins from the RVFV insect vector *Aedes aegypti*. Using an ELISA-based binding assay, we screened RVFV binding to CLRs from hosts, as well as insect vectors. The comparative data allowed us to directly compare CLR homologues and to identify species-related differences in the binding of CLRs. The found similarities and differences may help to better understand the virus coevolution with mosquito vector and mammalian host PRRs and may lead to opportunities to interfere with virus interactions with vector and host.

## 2. Results

### 2.1. Mosquito CLR–hFc Fusion Protein Expression

**Figure 1:**
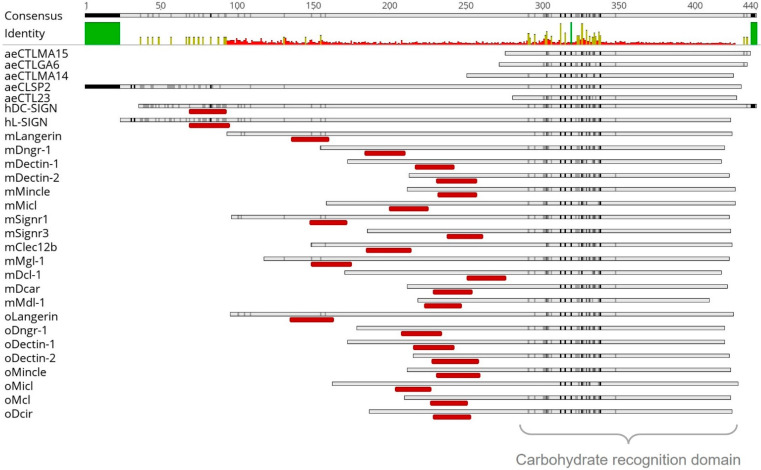
Alignment of 28 C-type lectin receptor amino acid sequences (ae: *Aedes aegypti*, h: *Homo sapiens sapiens*, m: *Mus musculus*, o: *Ovis aries)*. Amino acids similarity analysed with Score Matrix Blossum62 (Threshold 1) and symbolised in light grey (60–79% similarity), dark grey (80–99%), and black (100%). Hydrophobic part, indicating a transmembrane region, marked in dark red below the sequence. Above, consensus and mean pairwise identity over all amino acid sequences shown (green: 100% identity; yellow: 30–99%; red: <30%). Mosquito amino acid sequences were obtained from VectorBase and mammalian sequences from NCBI (Table 1 and Table 2). Sequence analysis was performed, and figures were prepared using the Geneious Prime software.

**Figure 2:**
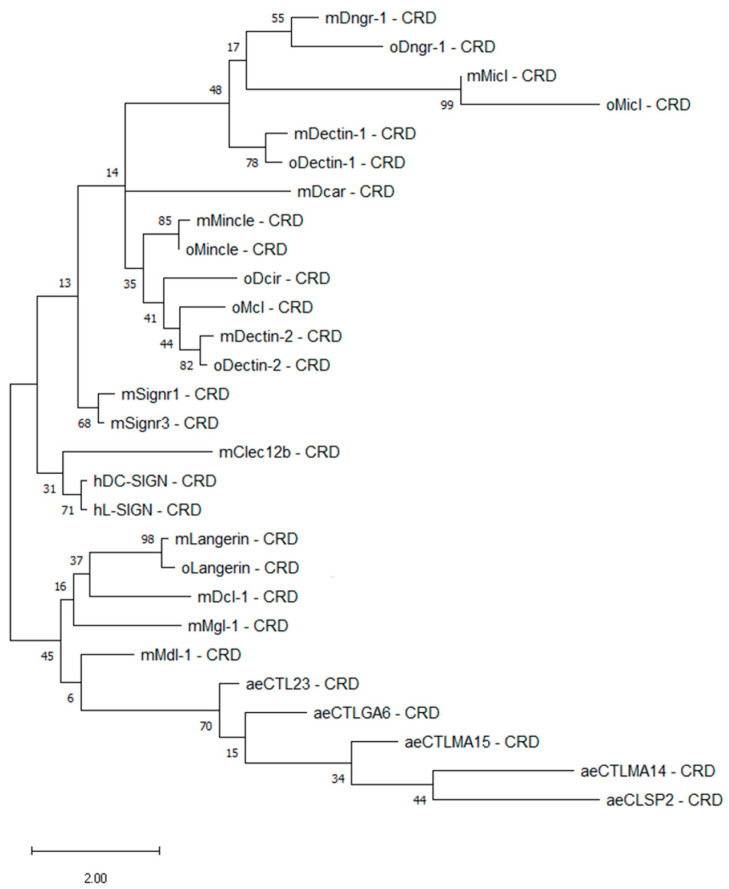
Maximum likelihood phylogenetic analysis of the carbohydrate recognition domain (CRD) of 28 C-type lectin receptors. Amino acid sequences of the respective CRDs were modelled by using bootstrap with 500 replications with the Jones–Taylor–Thornton plus frequency model and a discrete gamma distribution with five rate categories. Percentage frequencies from the bootstrap procedure are shown at each knot. Mosquito amino acid sequences were obtained from VectorBase, while mammalian sequences were from NCBI (Table 1 and Table 2). ae: *Aedes aegypti*, h: *Homo sapiens sapiens*, m: *Mus musculus*, o: *Ovis aries*. Phylogenetic analysis was performed with MEGA Software.

To evaluate RVFV–CLR interactions in a cross-species approach, mosquito vector CLR–hFc fusion proteins were expressed in addition to available murine and ovine CLR–hFc fusion protein libraries [33,34,35]. Initially, a maximum likelihood phylogenetic analysis of the CRDs was performed to compare the CLRs across the different species. In a recent publication, we showed that ovine, bovine, and caprine CLRs have higher degrees of morphological similarities to humans than to murine homologues [35]. In this study, we further compared the mammalian CLRs with mosquito CTLDcps. Alignment (Figure 1) and subsequent phylogenetic tree analysis (Figure 2) of the mammalian and mosquito CRD amino acid sequences confirmed that mammalian CLR homologues possessed highly conserved residues, while it has predicted with more than 70% accuracy that all five *Aedes* CTLDcps share close roots (Figure 2).

**Figure 3:**
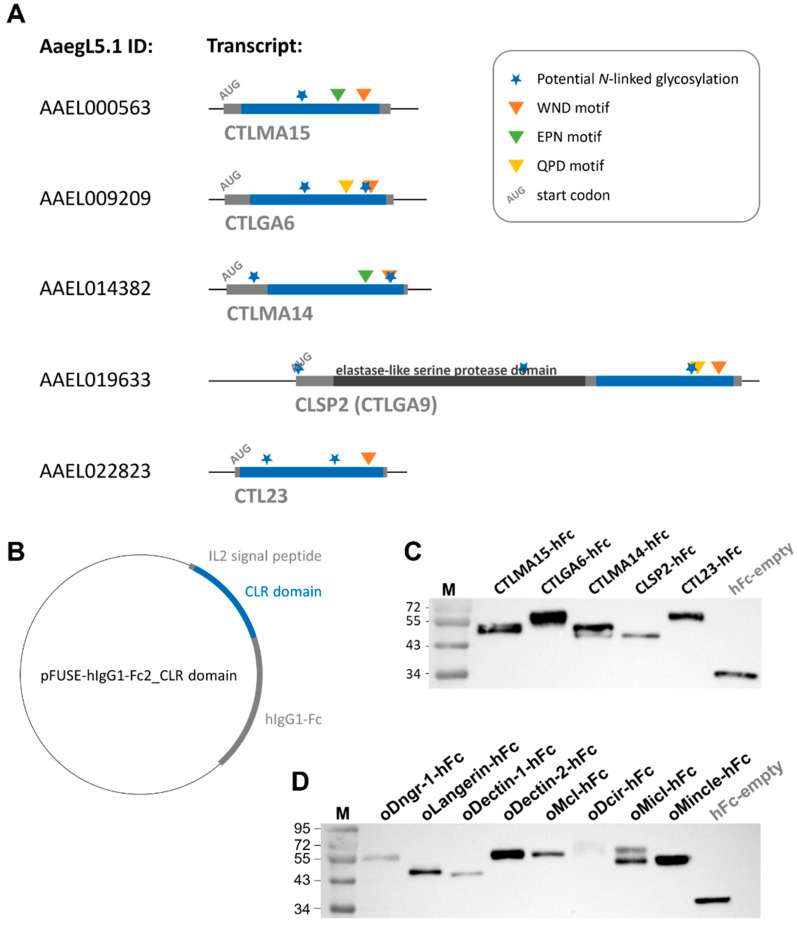
Mosquito CLR–hFc fusion proteins: (**A**) schematic overview of five *Aedes aegypti* transcripts (―) encoding for CTLDcps. Open reading frame is marked in grey, and nucleotides extracted for produced hFc–fusion proteins are highlighted in blue. Start codon and four conserved genetic motifs symbolised, as shown in the legend. AaedL5.1 CLR nucleotide sequences were obtained from VectorBase; (**B**) vector map of CLR domain ligated into pFUSE–hIgG1–Fc2 (Invitrogen); (**C**,**D**) Western blot image of anti-hFc stained mosquito (**C**) and ovine (**D**) CLR–hFc fusion proteins and hFc-empty control. Lane M: protein molecular standard, in kDa.

To identify the CRD of the mosquito CLR sequences, an alignment to known mammalian CLRs was performed (Figure 1). The CRD of CLRs comprised 110–130 amino acid residues, with conserved disulphide bonds and up to four Ca^2+^ binding sites [36]. This CRD could be found in all mammalian and mosquito proteins used in this study and showed high sequence similarities (Figure 1, Supplementary Figure S1). In comparison to the human, murine, and ovine myeloid CLRs, the selected mosquito proteins aeCTLMA15, aeCTLGA6, aeCTLMA14, aeCLSP2, and aeCTL23 did not show any hydrophilic part (Figure 1). To roughly estimate possible CTLDcps ligands, the CRD amino acid sequences were screened for conserved Ca^2+^ dependent glycan-binding residues. The WND motif (Trp–Asn–Asp), known for galactose and *N*-acetylgalactosamine (GalNAc) binding [36,37,38], was present at the C-terminal region of all five CTLDcps (Figure 3A; Supplementary Figure S1). Furthermore, aeCTLMA15 and aeCLTMA14 contained EPN motifs (Glu–Pro–Asn), described as a mannose-, *N*-acetylglucosamine (GlcNAC) and glucose-binding motif, while aeCTLGA6 and aeCLSP2 displayed the QPD motif known to interact with galactose (Gln–Pro–Asp) [37,38] (Figure 3A; Supplementary Figure S1). Additionally, all *Aedes aegypti* CLRs had one or more putative N-glycan sequons (Asn-X-Ser/Thr, where X can be any amino acid except proline [39,40]) (Figure 3A).

As the correct CRD folding and processing is crucial for subsequent analysis of CLR–hFc fusion protein–ligand interaction, the full CRD sequence with its potential glycan-binding sites and *N*-linked glycosylation motifs was ligated into the pFUSE–hIgG1–Fc2 vector (Figure 3A,B). After expression in CHO-S cells and protein purification, protein identity and purity were confirmed by SDS–PAGE and subsequent Western blot analysis. CLR–hFc fusion proteins were purified from cell culture supernatant and detected by anti-hFc staining (Figure 3C,D). The fact that some ovine and mosquito CLR–hFc fusion proteins showed two bands or had an apparently higher molecular weight than calculated based on their amino acid sequence (Table 1) may indicate the presence of different glycoforms [35], thereby increasing the protein mass. CLR–hFc fusion protein preparations were highly pure, as no further protein bands were detectable by protein staining (Supplementary Figure S2A,B).

### 2.2. ELISA-Based Binding Studies

**Figure 4:**
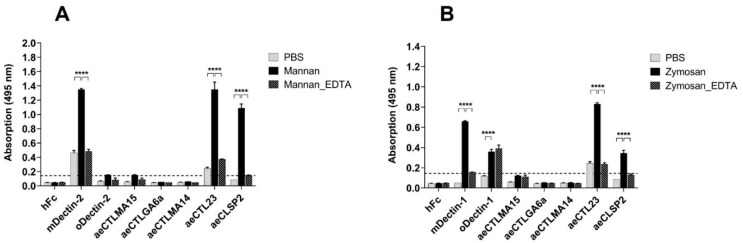
Mosquito CLR–hFc fusion protein interaction to mannan and zymosan: (**A**) ELISA-based screening of mannan with five mosquito CLR–hFc fusion proteins, in the presence and absence of EDTA. mDectin-2/oDectin-2 are known to recognise mannan (positive control) [35,41] and hFc-empty employed as negative control. One representative of *n* = 4 (EDTA *n* = 2) independent experiments shown; (**B**) ELISA-based screening of zymosan with five mosquito CLR–hFc fusion proteins, in the presence and absence of EDTA. mDectin-1/oDectin-1 are known to recognise zymosan (positive control) [35,43] and hFc-empty employed as negative control; (**A**,**B**) to discard possible false positives, the dotted line represents the cutoff defined as the threefold margin of the absorbance value relative to hFc, based on previous screenings with the CLR–hFc fusion protein library [34,46]. Data are depicted as mean + SEM of duplicates. One representative of *n* = 4 (EDTA *n* = 2) independent experiments is shown. One-way ANOVA, along with subsequent pairwise Tukey tests, was performed to compare the binding of the CLR–hFc fusion proteins above the threshold to PBS control and the EDTA supplementation each; **** *p* < 0.0001.

Given the observed CRD similarities between mosquito and mammalian CLRs, as well as the sugar-binding motifs in the amino acid sequence of mosquito CLRs, we hypothesised that known mammalian CLR ligands may be recognised by mosquito CLRs as well. Mannan, an α-1,6 linked mannose homopolysaccharide and a known Dectin-2/Langerin ligand [41,42], and zymosan, a β-1,3-linked glucose homopolysaccharide and Dectin-1 ligand [43,44], were coated on ELISA plates and probed with mosquito CLR–hFc fusion proteins. AeCTL23 and aeCLSP2 bound to both mannan and zymosan (Figure 4A,B). These interactions were abrogated by adding 10 mM ETDA to the binding buffer (Figure 4A,B), indicating Ca^2+^-dependent binding.

**Figure 5:**
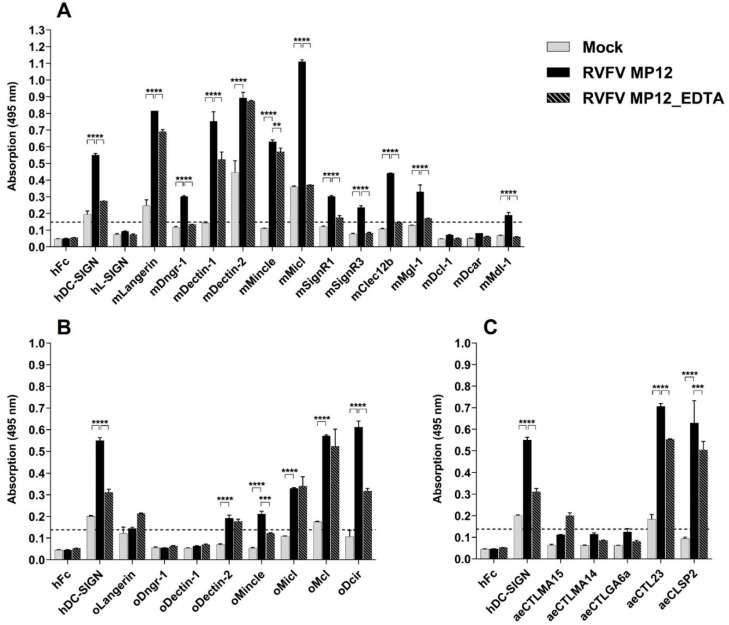
CLR–hFc fusion proteins across species bind to RVFV MP12: (**A**) ELISA-based binding study of purified RVFV MP12/Mock with murine CLR–hFc fusion proteins in the presence and absence of EDTA. hDC-SIGN served as positive control [24,45] and hFc-empty as negative control. One representative of *n* = 5 (EDTA: *n* = 2) independent experiments is shown; (**B**) ELISA-based screening of purified RVFV MP12/Mock with ovine CLR–hFc fusion proteins, in the presence and absence of EDTA. hDC-SIGN served as positive control and hFc-empty as negative control. One representative of *n* = 3 (EDTA: *n* = 2) independent experiments is shown; (**C**) ELISA-based screening of purified RVFV MP12/Mock with *Aedes aegypti* CLR–hFc fusion proteins, in the presence and absence of EDTA. hDC-SIGN served as positive control and hFc-empty as negative control. One representative of *n* = 3 (EDTA: *n* = 2) independent experiments is shown; (**A**–**C**) to discard possible false positives, the dotted line represents the cutoff defined as the threefold margin of the absorbance value relative to hFc, based on previous screenings with the CLR–hFc fusion protein library [34,46]. Data are depicted as mean + SEM of duplicates. One-way ANOVA with subsequent pairwise Tukey tests were performed to compare the binding of the CLR–hFc fusion proteins above the threshold to mock and the EDTA supplementation; ** *p* < 0.01, *** *p* < 0.0005, **** *p* < 0.0001.

To identify novel RVFV–CLR interactions, the purified virus was immobilised on the ELISA plate. The purified mock control served to determine the level of unspecific CLR–hFc fusion protein binding to remaining host cell proteins from the virus preparation. Human DC-SIGN was used as a positive control, as its role in RVFV recognition and host cell entry was already shown [24,45]. mLangerin, mDectin-1, mDectin-2, mMincle, and mMicl all showed increased absorption, compared with respective mock controls (Figure 5A). In the presence of EDTA, binding of the fusion proteins mMicl–hFc and hDC–SIGN–hFc to RVFV was abolished, while binding of mDectin–2-hFc and mMincle–hFc remained unaltered.

Ovine CLRs were tested in the same assay. While mLangerin–hFc and mDectin–1-hFc bound substantially to RVFV (Figure 5A), oLangerin–hFc and oDectin–1-hFc did not (Figure 5B). In summary, we could observe differences in the RVFV interaction of murine and ovine CLR homologues. Despite their similarities in their amino acid sequences (Figure 2), they exhibited differential ligand binding and varied in the calcium dependency of the interactions (Figure 5A,B). The ELISA-based screening thus identified novel ovine RVFV–CLR interactions such as oMcl and oDcir. To analyse RVFV–mosquito CTLDcps interactions, *Aedes aegypti* CLR–hFc fusion proteins were tested accordingly. Compared with the positive control hDC–SIGN–hFc [24,45] and respective mock controls, aeCTL23–hFc and aeCLSP2–hFc showed marked binding to RVFV (Figure 5C). These binding studies suggest that mosquito CTLDcps may be involved in RVFV recognition; however, their role in RVFV infection and immunity in mosquitoes needs to be further investigated.

## 3. Discussion

Myeloid CLRs play important roles in virus recognition, resulting in diverse immune responses or host cell entry [17]. In this study, we used murine and ovine CLR–hFc fusion proteins [33,34,35] to identify RVFV binding candidates and compared the binding of CLR homologues in a cross-species approach. Since only human DC-SIGN and L-SIGN were previously reported to interact with RVFV and subsequently facilitate viral host cell entry [24,45], the role of many mammalian CLRs in RVFV recognition has not been investigated so far. Murine Langerin, Dectin-1, Dectin-2, Mincle, and Micl (Clec12a) seem to be CLR-binding candidates for RVFV (Figure 5A). Except for Langerin, their role in viral recognition is largely unknown. Micl has been shown to interact with monosodium urate crystals [47,48], and plasmodial hemozoin [49], whereas Dectin-1 and Dectin-2 are well known to recognise fungal ß-glucans and α-mannans, respectively, and both contribute to host defence [41,43,44,50,51]. Only a limited number of publications highlight the role of these CLRs in viral recognition. Whole-blood transcriptome analysis identified Micl to be upregulated during SARS-CoV-2 infection in humans [52]. Dectin-2 senses influenza virus hemagglutinin, which initiates IL-12p40 and IL-6 production in murine bone marrow-derived dendritic cells [53]. In a previous study, we showed Mincle to interact with La Crosse virus; however, its role in early antiviral responses against this bunyavirus was limited in vitro [34]. Further approaches are needed for the validation of the identified CLR–RVFV interactions and for a functional characterisation to investigate their potential biological relevance. Thus, it remains to be determined whether Langerin, Dectin-1, Dectin-2, Mincle and/or Micl specifically interact with RVFV and/or contribute to antiviral responses.

Langerin (CD207), among those identified as RVFV-binding candidates, is expressed by resident dendritic cells of the epidermis, known as the so-called Langerhans cells [54]. Consequently, this CLR is present at the anatomical site of initial RVFV infection after a mosquito bite. Langerin has been reported to be a receptor preventing Langerhans cells from human immunodeficiency virus I infection by internalising this virion and subsequently initiating its degradation [55]. Furthermore, Langerin is an attachment and entry receptor for influenza A virus [56]. As Langerin is known as a viral receptor [55,56], and to recognise high-mannose glycans [57], which are also present on RVFV surface glycoproteins [58], this RVFV-binding candidate may be functionally involved in host RVFV recognition.

While soluble CLR–hFc fusion proteins are useful to screen for pathogen–CLR interactions, the mode of presentation of the CRD may markedly affect ligand recognition [19,34,35,46,59]. By using a library of dimeric CLR–hFc fusion proteins with the CRD N-terminally fused to the Fc fragment of human IgG1, we may miss RVFV interactions with CLRs for which a multimeric presentation is crucial, thereby leading to false-negative results. However, dimeric CLR–hFc fusion proteins have proven useful for initial screenings to identify novel CLR–pathogen interactions [19,34,35,46]. Dimeric DC-SIGN-hFc, for instance, also recognises low-affinity ligands in comparative screenings with monomeric and/or tetrameric DC-SIGN presentation [60,61].

All binding studies were performed with RVFV particles matured in the *Aedes albopictus* cell line C6/36. Thus, the viral particles are likely similar to RVFV from virus-containing saliva injected into the mammalian host. Previous publications already reported structural differences in the composition of envelope lipids [62,63], glycans [24,64], and even surface proteins [65] of arboviruses replicating in mammalian versus insect cells. Sindbis virus, for example, was shown to bind more efficiently to hDC-SIGN and hL-SIGN, when produced in insect cells [66]. Furthermore, experimental RVFV infection in goats varied in early viral replication and immune response when animals were infected with RVFV maturated in C6/36 cells versus Vero cells (African green monkey cells) [67]. Consequently, RVFV derived from insect cells may interact with CLRs in a different manner than mammalian cell-derived virions. Previous studies on the binding of RVFV to hDC-SIGN investigated the interaction of hDC-SIGN with RVFV maturated in BHK-21, Vero, or other mammalian cells [24,45,58]. Here, we showed that RVFV matured in mosquito cells was bound by hDC-SIGN as well. Thus, host CLRs may be involved in recognition of initial virus infection after mosquito bites but may further interact with RVFV proliferating in the host. Whether species-specific differences in glycosylation, envelope lipids, or surface protein composition of bunyaviruses have a direct impact on CLR binding and downstream signalling has to be further investigated in future studies.

Besides finding murine RVFV binding candidates, we could observe differences between the binding affinity of murine and ovine CLR homologues. Different ligands [68,69] and diverse functionalities between CLR homologues [69,70] were already described in the context of bacterial infections. Even though the Langerin sequence is highly conserved among mammalian species, human and murine homologues showed miscellaneous binding to numerous bacterial polysaccharides [68]. The binding site of both receptors is highly similar, but even subtle changes besides the glycan-binding site seemed to yield diverse ligand specificity [68], as was also reported for the closely related human CLRs DC-SIGN and L-SIGN [71]. Consequently, homology in protein sequences of CLRs does not necessarily result in similar ligand-binding preferences, as also seen in our cross-species CLR–RVFV binding study for ovine and murine CLR homologues. Moreover, CLR homologues can bind different ligands, and their downstream signalling pathways and effector functions can vary among different species as well [69,70,72,73].

As arboviruses such as RVFV circulate between insect vector and mammalian hosts, they are able to interact with very different host systems. On the one hand, they replicate in poikilothermic insects with innate cellular and humoral immune responses [74,75]; on the other hand, the same virus infects vertebrates and has to cope with their complex innate and adaptive immune mechanisms. Given that a virus only encodes for a limited number of structural proteins, the question remains as to how arboviruses can interact with the host and vector PRRs to maintain their cross-species transmission cycle. As RVFV is known to interact with host CLRs [24,45], and C-type lectin domain-containing proteins (CTLDcps) were described in *Aedes aegypti* [28], we hypothesised that RVFV may interact with these insect receptors as well. In total, 57 CTLDcps genes of *Aedes aegypti* are known so far [28]. In this study, we focused on five selected CTLDcps (CTLMA14, CTLMA15, CTLGA6, CTL23, and CLSP2), as their expression was upregulated after West Nile virus and/or Japanese encephalitis virus infection [29,30], suggesting a role in mosquito antiviral responses. While the CRD of mosquito CTLDcps deviate from mammalian CLRs, conserved motifs, known for calcium-dependent glycan-binding in myeloid CLRs, were found in all five mosquito CTLDcps. In our ELISA-based binding study, *Aedes aegypti* CTL23 (also named mosGCTL-11) and CLSP2 (also named CTLGA9) bound to α-1,6 linked mannose (mannan) and β-1,3 linked glucose (zymosan) in a calcium-dependent manner. This finding indicates that mosquito CTLDcps may recognise similar pathogen-associated molecular patterns such as mammalian CRLs, thereby highlighting the phylogenetic relevance of this PRR class for the innate recognition of viral pathogens. Whether these CRD motifs coevolved due to the high evolutionary pressure in host–pathogen interactions or remained unchanged since the latest common ancestor remains unanswered. Xia et al. hypothesised that insect CTLDcps have undergone species-specific expansion, as *Aedes aegypti*, *A. gamiae*, *A. pisum*, and further insect CTLDcps form species-related clusters in a cross-species phylogenetic analysis [76].

## 4. Materials and Methods

### 4.1. Cell Culture

Vero cells (clone E6, CCLV-RIE-929/25) were cultivated in MEM with Earle’s salts (Capricorn Scientific, Ebsdorfergrund, Germany) supplemented with 2 mM stable L-glutamine (Capricorn Scientific), 100 U/mL penicillin, 100 µg/mL streptomycin (Capricorn Scientific), and 10% fetal bovine serum (FBS) (Capricorn Scientific) at 37 °C in 5% CO_2_. C6/36 (CCLV-RIE-1299) cells were grown in Schneiders Drosophila media (PAN-Biotech, Aidenbach, Germany) supplemented with 2 mM stable L-glutamine (Capricorn Scientific), 1 mM sodium pyruvate (PAN Biotech), 1X MEM NEAA (PAN Biotech), 100 U/mL penicillin, 100 µg/mL streptomycin (Capricorn Scientific), and 10% FBS (Biowest, Riverside, MO, USA) at 28 °C. The suspension cell line FreeStyle™ CHO-S (Thermo Fisher Scientific, Waltham, MA, USA) was cultivated in FreeStyle^TM^ CHO medium (Thermo Fisher Scientific) supplemented with 8 mM stable L-glutamine (Capricorn Scientific), 100 U/mL penicillin, 100 µg/mL streptomycin (Capricorn Scientific) at 37 °C, and 5% CO_2_ on an orbital shaker.

### 4.2. Virus Cell Culture and Purification

Rift Valley fever virus strain MP12 was produced in seven T-175 flasks by infecting 80% confluent C6/36 cells with a multiplicity of infection (MOI) of 1. Mock infection was performed in the exact same manner, by cultivating seven T-175 flasks of uninfected C6/36. Virus containing supernatant as well as mock infection supernatant were collected at 3 dpi, pooled, and cleared by centrifugation (1200× *g*; 20 min; 4 °C). Afterwards, supernatants were concentrated and cleared from host-cell-derived proteins via ultracentrifugation in an Optima XPN (Beckman Coulter, Brea, CA, USA) using SW32Ti and SW60Ti rotors (Beckman Coulter). This RVFV purification method was adapted from a protocol published for UUKV concentration [77]. First, 35 mL virus or mock supernatant were filled into a 38.5 mL polyallomer centrifuge tube (Seton scientific, Petaluma, CA, USA) and layered under with 3 mL of 25% sucrose solution in 1× HNE buffer (10 mM HEPES, 150 mM NaCl, 1 mM EDTA, pH 7.3). The centrifugation occurred at 96,000× *g* and 4 °C for 2 h. Formed pellets were resuspended in 1× HNE buffer. A sucrose density gradient centrifugation was performed to further purify the virus and remove host-cell-derived proteins. In a 4.4 mL clear ultracentrifuge tube (Seton scientific), 600 mL of 60%, 45%, 30%, and 15% sucrose solution in 1× HNE were layered from highest to lowest density. After adding 1.5–2.0 mL virus or mock solution, the density gradient centrifugation was performed at 96,000× *g* and 4 °C for 90 min with deceleration set on minimum. The arising virus band and mock at the same density were collected. To further clear the virus from the remaining sucrose, the virus band was transferred into a 4.4 mL clear ultracentrifuge tube (Seton Scientific), 0.5 mL of 25% sucrose solution was underlayered and centrifuged at 96000× *g* and 4 °C for 90 min. The arising pellet was resuspended in 250 µL 1× HNE buffer and stored at −80 °C. The 50% tissue culture infective dose (TCID50) was determined using Vero E6 cells. Plaque forming units (PFU/mL) were estimated using the formula 0.69 × TCID50, as described previously [78].

### 4.3. CLR–hFc Fusion Protein Production

Human, murine, and ovine CLR–hFc fusion proteins were produced, as described earlier [33,34,35]. Following these protocols, the mosquito CTLDcps were transiently expressed as chimeric hFc fusion proteins. In short, proteinogenic sequences of each mosCTLDcps, obtained from VectorBase AaegL5.1 genome assembly (AaegL5.1 IDs shown in Table 1), were synthesised by Eurofins Genomics (Ebersberg, Germany) and served as template DNA. To define the CRD, mosquito CTLDcps were aligned with known murine and ovine CLRs (Figure 1). The carbohydrate recognition domain (CRD) and the C-terminal parts of the proteins were amplified by PCR (primers shown in Table 1; amplified region shown in Figure 3A) and ligated into the pFUSE–hIgG1–Fc2 expression vector (InvivoGen, Toulouse, France). The sequences were verified by Sanger sequencing with a Mix2seq Kit (Eurofins Genomics) according to the manufacturer’s protocol. For protein expression, the pFUSE–hIgG1–Fc2 plasmids encoding the CRDs (Figure 3B) were transiently transfected with 25 kDa linear polyethylenimine (Polysciences, Warrington, PA, USA) into FreeStyle™ CHO-S cells (Thermo Fisher Scientific). The negative control (hFc empty) was produced in the exact same manner, by transfecting the empty pFUSE–hIgG1–Fc2 plasmid. After 96 h, secreted fusion proteins were purified from cell supernatant using HiTrap protein G affinity chromatography columns (GE Healthcare, Danderyd, Sweden) and final protein concentrations were calculated using a Micro BCA™ Protein Assay Kit (Thermo Fisher Scientific) according to the manufacturer’s protocol.

### 4.4. Western Blot

Western blot and ROTI^®^-Blue staining of SDS-page gels were performed to verify the size, integrity, and purity of the produced fusion proteins. A total of 0.3 µg of each protein was separated in denaturing SDS–PAGE gel electrophoresis and transferred to nitrocellulose membrane. After blocking with milk powder overnight and staining with goat anti-human IgG-horseradish peroxidase antibody (Dianova, Hamburg, Germany) for 1 h, the membrane was covered with SuperSignal^TM^ West Dura solution (Thermo Fisher Scientific) as described in the manufacturer’s manual. Chemiluminescence was detected using a ChemiDoc^TM^ MP System (Bio-Rad Laboratories, Hercules, CA, USA). For purity control, the gel was stained with ROTI^®^-Blue (Carl Roth, Karlsruhe, Germany) overnight and imaged using a ChemiDoc^TM^ MP System.

### 4.5. ELISA-Based RVFV MP12–CLR Binding Studies

Overnight, wells of a medium-binding half-area 96-well plate (Greiner Bio-one GmbH, Frickenhausen, Germany) were coated with either 1 µg of mannan (Sigma-Aldrich, MO, USA) or zymosan (Sigma-Aldrich) in 50 µL PBS, or 50 µL of 1 × 10^8^ PFU/mL purified RVFV MP12 or mock (see Section 2.2. Virus Cell Culture and Purification). The coated wells were washed three times, each with 150 µL of washing buffer (1X PBS, 0.05% Tween-20), and then blocked with 150 µL of 1% BSA (fraction V, IgG free, fatty acid poor, Thermo Fisher Scientific, Darmstadt, Germany) in 1X PBS for 2 h at room temperature, to prevent unspecific binding. The plate was again washed, followed by the addition of 0.25 ng/well fusion proteins diluted in 50 µL lectin binding buffer (50 mM HEPES, 5 mM MgCl_2_, 5 mM CaCl_2_, pH 7.4) for one hour. To test for calcium dependency, the fusion proteins were diluted in EDTA buffer (10 mM EDTA, 50 mM HEPES, pH 7.4) instead of lectin binding buffer. The plate was washed again, and 50 µL of anti-human IgG-horseradish peroxidase (HRP) antibody (Dianova, Hamburg, Germany) diluted 1:5000 in 1X PBS with 1% BSA and 0.05% Tween-20 was added. After one hour of incubation, the plate was finally washed before adding 50 µL of substrate solution (O-phenylenediamine dihydrochloride substrate tablet (Thermo Fisher Scientific), 24 mM citrate buffer, 50 mM phosphate buffer, and 0.04% H_2_O_2_). After 5 min of colour development, the reaction was stopped with 2.5 M sulfuric acid, and absorbance was measured at 495 nm using a Multiskan GO microplate spectrophotometer (Thermo Fisher Scientific).

### 4.6. Statistical and Phylogenetic Analysis

Nucleotide and amino acid sequence analyses, as well as their alignment and primer design, were performed using Geneious prime 2020.1.2 software (San Diego, CA, USA). Biophysical properties of CRL–hFcs, such as protein molecular weight and transmembrane domain position, were assessed using the Sequence Manipulation Suite [79] and TMHMM–2.0 [80,81] respectively. The phylogenetic analysis was carried out with MEGA 11.0.10 software (www.megasoftware.net, 13 December 2021) and with 500 bootstrap replicates. Protein and nucleotide sequences were obtained from the National Centre for Biotechnology Information (NCBI) genome database (www.ncbi.nlm.nih.gov/genome, last access date: 13 December 2021) or from VectorBase (www.vectorbase.org, last access date: 13 December 2021) (Table 1 and Table 2). ELISA data plots were generated with GraphPad Prism 7 software (San Diego, CA, USA), and metric data are represented as a mean + SEM (standard error of mean) for all experiments.

## 5. Conclusions

In this study, we identified candidate CLRs from human, mouse, sheep, and *Aedes aegypti* binding to RVFV. Furthermore, species-specific differences in CLR homologues in RVFV binding were observed. Our findings may present a first step towards a better understanding of virus–CLR interactions across species and may help to develop novel strategies for interfering with such interactions.

## Figures and Tables

**Table 1:** Primers (fw, forward; rev, reverse) used to amplify the extracellular domain of mosquito C-type lectin domain-containing proteins (CTLDcps) from reference genome AaegL5.1 (VectorBase). Terminal restriction sites are underlined, and primer binding sites are shown in italic. Respective PCR amplicon size in base pairs (bp) and final CLR–hFc fusion protein size (after cleavage of IL-2 signal peptide after Ser20) in kilodaltons (kDa) are indicated.

CTLDcps	AaegL5.1 ID	Primer Sequences (5′-3′)	Amplicon Size	CLR–hFc Fusion Protein Size
aeCTLMA15	AAEL000563	fw: GATATCA*TCTCATGGAGATTCTACGCC* rev: CCATGGT*CTCGCATATGAAATACAGCG*	413 bp	41.62 kDa
aeCTLGA6	AAEL009209	fw: GAATTCC*TGTACCTCGCCATCG* rev: CCATGGT*CTCACAAACCGGCACC*	407 bp	41.43 kDa
aeCTLMA14	AAEL0114382	fw: GAATTCG*TGTCGATGTGAAGCGG* rev: CCATGGT*TTCACAAACGAATTTCAATC*	407 bp	41.3 kDa
aeCLSP2	AAEL019633	fw: GAATTCA*TGCTTACATCAGCC* rev: CCATGGT*TTCACAAATGTAGCG*	410 bp	41.28 kDa
aeCTL23	AAEL022823	fw: GAATTCG*GCACCTAGCTTGGTC* rev: CCATGGT*TTCACAGATAAAATACTTCTTC*	428 bp	41.82 kDa

**Table 2:** National Centre for Biotechnology Information (NCBI) Protein IDs, as well as gene name for each human, murine, and ovine C-type lectin used for phylogenetic analysis listed.

C-Type Lectin	Gene	Protein ID	C-Type Lectin	Gene	Protein ID
**hDC-SIGN**	CD209	NP_066978.1	**mMincle**	Clec4e	NP_064332.1
**hL-SIGN**	CLEC4M	NP_055072.3	**mSignr1**	Cd209b	NP 081248.4
**mClec12b**	Clec12b	NP_001191152.1	**mSignr3**	Cd209d	NP 570974.1
**mDcar**	Clec4b1	NP_001177239.1	**oDcir**	Clec4A	XP_042103517.1
**mDcl1**	Clec2i	NP_001276635.1	**oDectin-1**	Clec7A	XP_042103479.1
**mDectin-1**	Clec7a	NP_064392.2	**oDectin-2**	Clec6A	XP_004006949.1
**mDectin-2**	Clec6a	NP_064385.1	**oDngr-1**	Clec9A	XP_004006925.4
**mDngr-1**	Clec9a	NP_001192292.1	**oLangerin**	Clec4K, CD207	XP_004006101.3
**mLangerin**	Clec4k, CD207	NP_659192.2	**oMcl**	Clec4D	XP_042103518.1
**mMdl-1**	Clec5a	NP_001033693.1	**oMicl**	Clec12A	XP_004006929.1
**mMgl-1**	Clec10a	NP_001191181.1	**oMincle**	Clec4E	XP_042103520.1
**mMicl**	Clec12a	NP_808354.1			

## Data Availability

The data presented in this study are available on request from the corresponding authors.

## Supplementary Materials

Supplementary materials can be found at https://www.mdpi.com/article/10.3390/ijms23063243/s1.
