# Supplementary material for: Vector and Host C-Type Lectin Receptor (CLR)–Fc Fusion Proteins as a Cross-Species Comparative Approach to Screen for CLR–Rift Valley Fever Virus Interactions
Source: Int J Mol Sci. 2022 Mar 17;23(6):3243. doi: 10.3390/ijms23063243 (PMC8954825; doi:10.3390/ijms23063243)
Supplement: Supplementary file 1 [file ijms-23-03243-s001.zip › ijms-1603366-supplementary.pdf]

International Journal of *Molecular Sciences*

MDPI

Supplementary Figures

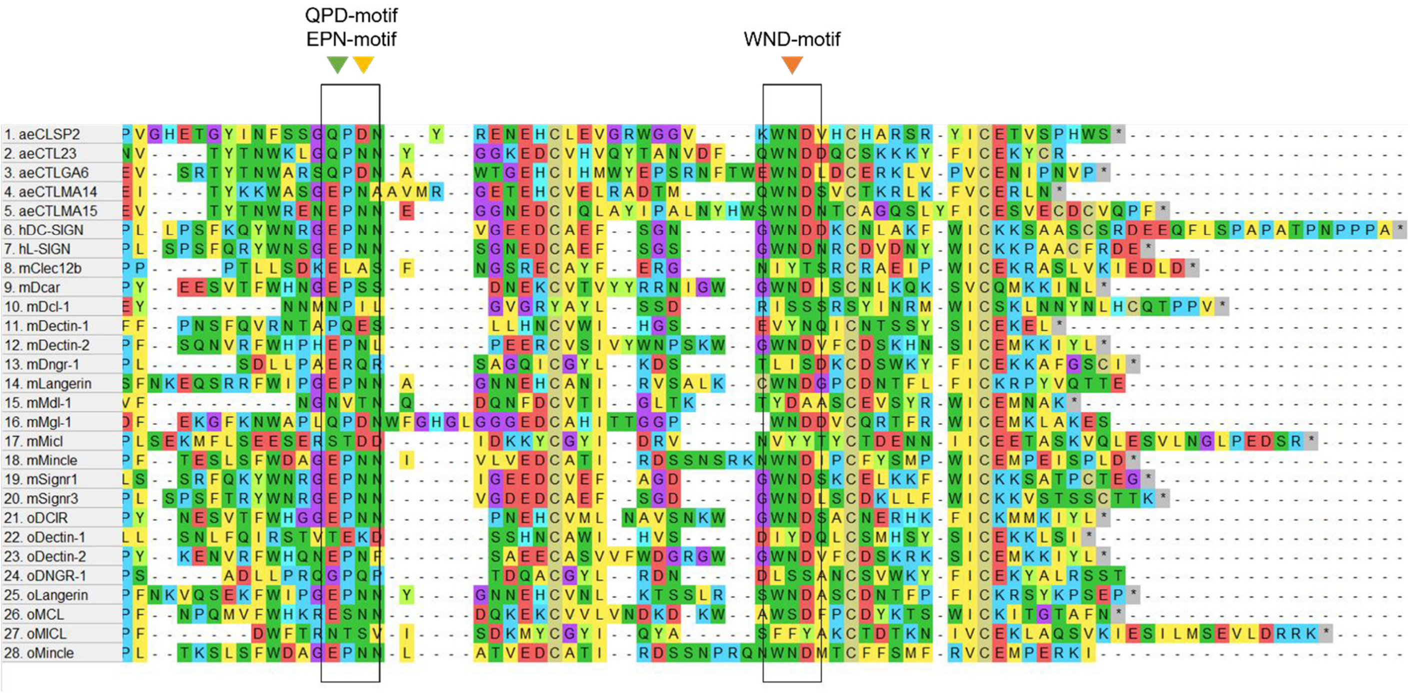

Figure S1. Alignment of the C-terminus of 28 C-type lectin receptors. Amino acids sequence alignment created with MEGA software (Muscle Alignment) of full CLR proteins, while only C-terminus of the CRD is shown here. WND, QPD, and EPN motifs are highlighted. ae: *Aedes aegypti*, h: *Homo sapiens sapiens*, m: *Mus musculus*, o: *Ovis aries*. Mosquito amino acid sequences were downloaded from VectorBase, while mammalian sequences were derived from NCBI (IDs shown in Tables 1 and 2).

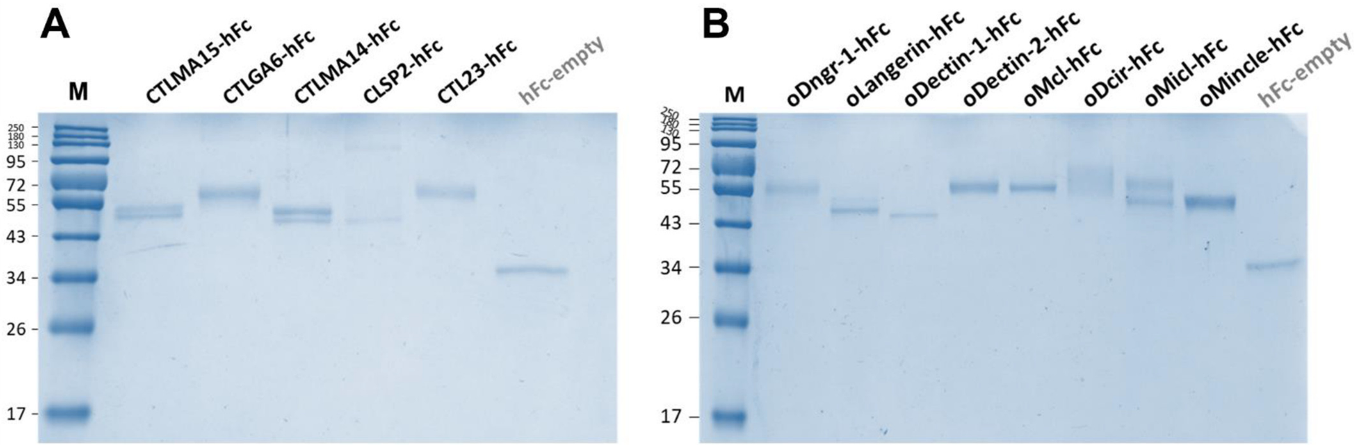

Figure S2. Purity of mosquito and ovine CLR–hFc fusion proteins: Mosquito (**A**) and ovine (**B**) CLR–hFc fusion proteins, as well as hFc-empty control separated by gel electrophoresis and stained with Roti BLUE®. Lane M: protein molecular standard, in kDa.
